# Comparative Analysis of Two Methods for the Detection of *EGFR* Mutations in Plasma Circulating Tumor DNA from Lung Adenocarcinoma Patients

**DOI:** 10.3390/cancers11060803

**Published:** 2019-06-10

**Authors:** Ming-Szu Hung, Jr-Hau Lung, Yu-Ching Lin, Yu-Hung Fang, Shu-Yi Huang, Yuan-Yuan Jiang, Meng-Jer Hsieh, Ying-Huang Tsai

**Affiliations:** 1 Department of Pulmonary and Critical Care Medicine, Chang Gung Memorial Hospital, Chiayi branch 61363, Taiwan; m13@seed.net.tw (M.-S.H.); lin0927@cgmh.org.tw (Y.-C.L.); 8902062@cgmh.org.tw (Y.-H.F.); 8802022@cgmh.org.tw (S.-Y.H.); st40339@cgmh.org.tw (Y.-Y.J.); mengjer@cgmh.org.tw (M.-J.H.); 2 Department of Medicine, College of Medicine, Chang Gung University, Taoyuan 33302, Taiwan; 3 Department of Respiratory Care, Chang Gung University of Science and Technology, Chiayi Campus, Chiayi 61363, Taiwan; 4 Department of Medical Research, Chang Gung Memorial Hospital, Chiayi branch 61363, Taiwan; jrhaulung@gmail.com; 5 Department of Respiratory Care, College of Medicine, Chang Gung University, Taoyuan 33302, Taiwan; 6 Department of Pulmonary and Critical Care Medicine, Chang Gung Memorial Hospital, Linkou branch 33305, Taiwan

**Keywords:** lung cancer, EGFR, TKI, ctDNA, ARMS, MassARRAY

## Abstract

Mutations in the epidermal growth factor receptor (*EGFR*) are associated with various solid tumors. This study aimed to compare two methods for the detection of *EGFR* mutations in circulating tumor DNA (ctDNA) from lung adenocarcinoma (LUAD) patients and to evaluate the clinical significance of *EGFR* mutations in ctDNA. In this prospective cohort study, the *EGFR* mutation status of 77 patients with stage IIIB or IV LUAD was first determined using lung cancer tissue. The amplification refractory mutation system (ARMS) and single allele base extension reaction combined with mass spectroscopy (SABER/MassARRAY) methods were also used to detect *EGFR* mutations in plasma ctDNA from these patients and then compared using the *EGFR* mutation status in lung cancer tissue as a standard. Furthermore, the relationship between the presence of *EGFR* mutations in ctDNA after receiving first-line EGFR-tyrosine kinase inhibitor (EGFR-TKI) therapy and survival was evaluated. The overall sensitivity and specificity for the detection of *EGFR* mutations in plasma ctDNA by ARMS and SABER/MassARRAY were 49.1% vs. 56% and 90% vs. 95%, respectively. The agreement level between these methods was very high, with a kappa-value of 0.88 (95% CI 0.77–0.99). Moreover, 43 of the patients who carried *EGFR* mutations also received first-line EGFR-TKI therapy. Notably, patients with *EGFR* mutations in plasma ctDNA had significantly shorter progression-free survival (9.0 months, 95% CI 7.0–11.8, vs. 15.0 months, 95% CI 11.7–28.2; *p* = 0.02) and overall survival (30.6 months, 95% CI 12.4–37.2, vs. 55.6 months, 95% CI 25.8–61.8; *p* = 0.03) compared to those without detectable *EGFR* mutations. The detection of *EGFR* mutations in plasma ctDNA is a promising, minimally invasive, and reliable alternative to tumor biopsy, and the presence of *EGFR* mutations in plasma ctDNA after first-line EGFR-TKI therapy is associated with poor prognosis.

## 1. Introduction

The epidermal growth factor receptor (EGFR) pathway plays an important role in the growth, proliferation, and survival of various solid tumors, including non-small cell lung cancer (NSCLC) [1]. EGFR is, therefore, an important potential target for lung cancer therapy. Notably, some NSCLC patients carry activating mutations in the tyrosine kinase domain of *EGFR* and, unlike the majority of NSCLC patients, exhibit a favorable clinical response to EGFR-tyrosine kinase inhibitor (EGFR-TKI) therapy [2].

While mutations in *EGFR* have been found in less than 10% of non-Asian NSCLC patients, up to 30% of East Asian NSCLC patients carry such mutations [3]. Interestingly, most of these mutations were limited to exons 18–21 [4], and were most frequently detected in patients with lung adenocarcinoma (LUAD) [5]. Exon 19 deletions and exon 21 missense mutations are common *EGFR* activating mutations, and among these, exon 19 in-frame deletions and the L858R exon 21 missense mutation have been shown to represent approximately 80% of the EGFR-TKI-sensitive mutations in NSCLC [6]. Furthermore, several clinical trials have demonstrated that, in NSCLC patients, exon 19 deletions and exon 21 missense mutations were associated with a favorable response to first-line treatment with EGFR-TKIs, including gefitinib [7], erlotinib [8], and afatinib [9], compared to conventional chemotherapy. Importantly, another missense mutation, T790M in exon 20, is associated with EGFR-TKI resistance and has been detected in 30%–50% of the patients that initially responded to EGFR-TKI therapy but eventually acquired EGFR-TKI resistance. However, recent evidence indicates that osimertinib, a third-generation EGFR-TKI, can overcome T790M-mediated resistance to first- and second-generation EGFR-TKIs.

Liquid biopsy is a promising technique for cancer diagnosis and treatment and consists of the detection and isolation of circulating tumor cells, circulating tumor DNA (ctDNA), and exosomes as a source of genomic and proteomic information in patients with cancer [10]. In patients with lung cancer, different methods have been used successfully to detect *EGFR* mutations from ctDNA, and studies have demonstrated that this approach was valuable for diagnosis, predicting treatment response, and monitoring acquired therapy resistance [11,12]. While the amplification refractory mutation system (ARMS) method has been used to detect *EGFR* mutations in both lung cancer tissues [13] and plasma ctDNA [14], a single allele base extension reaction combined with mass spectrometry (SABER/MassARRAY) has also been used to detect the T790M *EGFR* mutation in plasma ctDNA [15]. However, the respective performances of the ARMS and SABER/MassARRAY methods for the clinical detection of *EGFR* mutations from plasma ctDNA have rarely been compared.

In this study, we determined the *EGFR* mutation status of LUAD patients using lung cancer tissues and compared the efficiency of the ARMS and SABER/MassARRAY methods in detecting *EGFR* mutations in ctDNA isolated from the plasma of these patients. The relationship between the *EGFR* status and clinical outcomes of LUAD patients who received first-line EGFR-TKI therapy was also evaluated.

## 2. Materials and Methods

### 2.1. Patients and Study Design

Between February 2013 and March 2017, 77 LUAD patients (57 with and 20 without *EGFR* mutations) were enrolled in this prospective cohort study of *EGFR* mutation detection in plasma ctDNA. At the start of the study, all patients were treatment-naive with stage IIIB or IV advanced LUAD, according to the 7th Edition of the American Joint Committee on Cancer (AJCC) staging system. Mutations in the *EGFR* gene were detected by ARMS, using the therascreen EGFR RGQ PCR kit (Qiagen, Hilden, Germany) according to the manufacturer’s recommendations, or SABER/MassARRAY, using the OncoFOCUS™ Panel v1.0 (Agena Bioscience, San Diego, CA, USA) with the MassARRAY system (Agena Bioscience), as previously described [15]. The *EGFR* mutations examined in this study included exon 19 deletions and the T790M and L858R missense mutations. 

The clinical variables including sex, age, smoking status, pathology, EGFR-TKIs, mutations, and stages of these patients were analyzed. Patients were treated with either gefitinib (250 mg/day), erlotinib (150 mg/day), or afatinib (40 mg/day) until progressive disease (PD) occurred. Three months after treatment initiation, the response to therapy was evaluated according to the Response Evaluation Criteria in Solid Tumors (RECIST) version 1.1 [16], following examination of target lesions by a chest computed tomography (CT) scan, brain magnetic resonance imaging (MRI), or bone scan. Progression-free survival (PFS) was defined as the time from the first treatment to PD or death from any cause, and overall survival (OS) was defined as the time from the diagnosis to death from any cause or surviving patients being censored at their last follow-up.

This study was approved by the Ethics Review Board of Chang Gung Memorial Hospital (IRB No. 201600915B0 and 201101405B0).

### 2.2. DNA Extraction

For *EGFR* mutation status analysis in lung cancer tissues, DNA was extracted from 5 μm-thick sections of formalin-fixed paraffin-embedded (FFPE) tumors using the QIAamp DNA FFPE Tissue Kit (Qiagen), according to the manufacturer’s recommendations.

For *EGFR* mutation status analysis in plasma ctDNA, blood samples were collected in ethylenediaminetetraacetic acid (EDTA) tubes after a lung cancer diagnosis or EGFR-TKI treatment failure. Then, ctDNA was purified from 1 mL of plasma using the QIAamp Circulating Nucleic Acid Kit (Qiagen) according to the manufacturer’s recommendations.

### 2.3. Statistical Analysis

All statistical analyses were performed using the MedCalc version 15 software (MedCalc Software, Ostend, Belgium). Survival was analyzed using the Kaplan–Meier estimator and log-rank test. The agreement level between the ARMS and SABER/MassARRAY methods was evaluated using the inter-rater agreement (kappa-value) test. The difference between ARMS and SABER/MassArray was compared by pairwise comparison of receiver operating characteristic curve (ROC) curves. The difference of tumor size was compared by *t*-test. A *p*-value < 0.05 was considered to be statistically significant.

## 3. Results

### 3.1. Comparison of ARMS and SABER/MassARRAY for the Detection of EGFR Mutations in Plasma ctDNA

**Figure 1:**
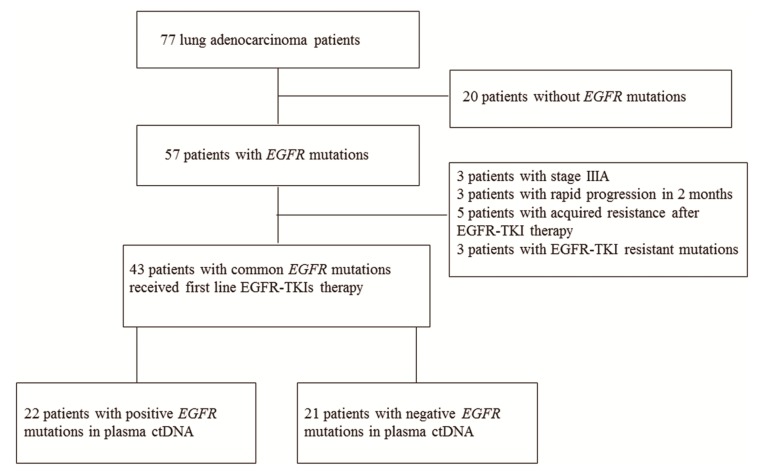
Flow chart of the study.

In order to compare the efficiency of ARMS and SABER/MassARRAY in detecting *EGFR* mutations in plasma ctDNA, we first determined the *EGFR* mutation status of the 77 LUAD patients enrolled in our study using the ARMS method. Using DNA isolated from lung cancer tissue, *EGFR* mutations were detected in 57 patients, whereas the other 20 patients carried only wild-type (WT) *EGFR* alleles (Figure 1). Three patients in our study demonstrated uncommon EGFR mutations (2 with G719X+S768I, and 1 with S768I) and without T790M, exon 19 deletions or L858R mutations in the lung cancer tissues. Next, we used ARMS and SABER/MassARRAY to detect the presence of *EGFR* mutations in the ctDNA isolated from the plasma of 77 and 70 patients, respectively.

Using the *EGFR* mutation status established from the lung cancer tissue as the standard, the overall sensitivity, specificity, and positive predictive value (PPV) of the ARMS method were 49.1%, 90%, and 93.3%, respectively (Table 1). In comparison, the overall sensitivity, specificity, and PPV of the SABER/MassARRAY method were 56%, 95%, and 96.6%, respectively (Table 1). Importantly, the level of agreement between the two methods was very high, with a kappa-value of 0.88 (95% confidence interval (CI) 0.77–0.99).

Focusing on *EGFR* activating mutations, the sensitivity, specificity, and PPV for the detection of exon 19 deletions by ARMS and SABER/MassARRAY were 50% vs. 53.8%, 95.6% vs. 97.7%, and 88.9% vs. 93.3%, respectively (Table 2). For the detection of the L858R missense mutation by ARMS and SABER/MassARRAY, the sensitivity, specificity, and PPV were 45% vs. 47.4%, 100% vs. 100%, and 100% vs. 100%, respectively (Table 3).

Moreover, a de novo T790M mutation, which is associated with EGFR-TKI resistance, was detected in the plasma ctDNA of one patient, while the T790M mutation was also detected in the re-biopsy samples of five patients following EGFR-TKI therapy failure. The sensitivity, specificity, and PPV for the detection of the T790M mutation by ARMS and SABER/MassARRAY were 33.3% vs. 50%, 100% vs. 100%, and 100% vs. 100%, respectively (Table 4). The difference between ARMS vs. SABER/MassARRAY were compared by a pairwise comparison of ROC curves and were not statistically significant for EGFR (*p* = 0.0687), L858R (*p* = 1.0000), Exon 19 deletions (*p* = 0.1708), and T790M (*p* = 0.3173) mutations.

### 3.2. Relationship between the EGFR Mutation Status after EGFR-TKI Therapy and Survival

In this study, we also evaluated the correlation between the presence of common *EGFR* mutations and the outcomes for patients who received EGFR-TKI therapy. Out of the 77 patients enrolled in the study, 43 carried common *EGFR*-activating mutations (L858R missense mutation and exon 19 deletions) detected by ARMS and received first-line EGFR-TKI therapy (Table 5). 

**Figure 2:**
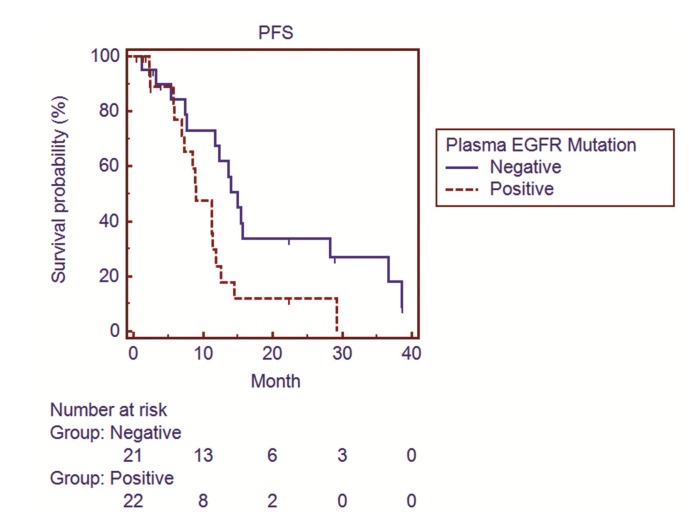
Survival curves showing the progression-free survival (PFS) of lung adenocarcinoma patients with and without detectable epidermal growth factor receptor (*EGFR*) mutations in plasma circulating tumor DNA (ctDNA) by ARMS.

**Figure 3:**
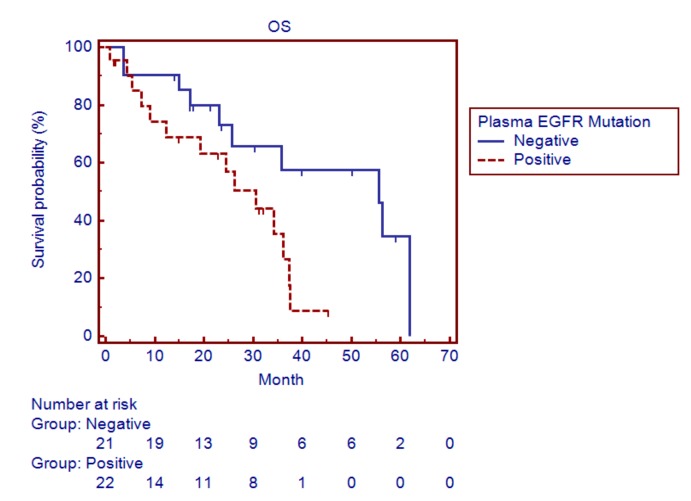
Survival curves showing the overall survival (OS) of lung adenocarcinoma patients with and without detectable epidermal growth factor receptor (*EGFR*) mutations in plasma ctDNA by ARMS.

Among these 43 patients, the presence of *EGFR* mutations in plasma ctDNA was significantly associated with shorter progression-free survival (PFS) (9.0 months for patients with detectable *EGFR* mutations, 95% CI 7.0–11.8, vs. 15.0 months for patients without detectable *EGFR* mutations, 95% CI 11.7–28.2; *p* = 0.02) (Figure 2) and OS (30.6 months for patients with detectable *EGFR* mutations, 95% CI 12.4–37.2, vs. 55.6 months for patients without detectable *EGFR* mutations, 95% CI 25.8–61.8; *p* = 0.03) (Figure 3).

In addition, common *EGFR* mutations were detected by SABER/MassArray in 37 of the 43 patients. Among these 37 patients, the presence of *EGFR* mutations in plasma ctDNA was associated with an insignificant trend for shorter PFS (11.2 months for patients with detectable *EGFR* mutations, 95% CI 7.0–12.5, vs. 14.0 months for patients without detectable *EGFR* mutations, 95% CI 7.6–38.5; *p* = 0.28) (Figure S1) and OS (30.6 months for patients with detectable *EGFR* mutations, 95% CI 12.4–61.8, vs. 35.7 months for patients without detectable *EGFR* mutations, 95% CI 17.3–61.8; *p* = 0.71) (Figure S2). 

Blood was collected from nine patients upon the diagnosis of lung cancer and 34 patients after EGFR-TKI therapy. The sizes of tumors at the time of the detection of plasma *EGFR* mutations were also evaluated. In 43 patients, regarding prognosis evaluation, the size of tumors at the time of the detection of *EGFR* mutations from plasma ctDNA were measurable using chest CT in 34 patients for ARMS and 30 patients for SABER/MassARRAY. Among these 34 patients, 12 patients showed decreased tumor size, 15 patients showed unchanged tumor size and, 7 patients showed increased tumor size at the time of blood collection. The average tumor size was significantly larger in patients with detectable plasma *EGFR* mutations compared to those without detectable plasma *EGFR* mutations (ARMS, 62.8 mm, 95% CI 43.1–82.5 mm vs. 32.8 mm, 95% CI 22.3–43.2 mm, *p* < 0.05; SABER/MassARRAY, 62.1 mm, 95% CI 40.3–83.9 mm vs. 31.4 mm, 95% CI 20.2–42.6 mm, *p* < 0.05) (Figure S3A,B).

## 4. Discussion

In this study, we demonstrated that both ARMS and SABER/MassARRAY are efficient methods for the detection of *EGFR* mutations in plasma ctDNA of LUAD patients. Furthermore, the two methods showed great potential for the clinical assessment of LUAD patients, exhibiting strong inter-rater agreement, high specificity, and high PPV. Our analyses also indicated that the presence of *EGFR* mutations in plasma ctDNA after first-line EGFR-TKI therapy was associated with poor prognosis.

A Qiagen therascreen EGFR RGQ PCR kit (ARMS method) has been approved in the United States as well as Europe and Asian countries to detect *EGFR* mutations with high sensitivity and specificity in lung cancer tissues [13]. Our results demonstrated that this kit could also be used reliably to detect *EGFR* mutations in plasma ctDNA isolated from lung cancer patients. Importantly, in our study, the observed sensitivity and specificity of ARMS were comparable to those reported in previous studies (sensitivity between 43.1% and 85.7%, with high specificity) [14,17,18]. Although our observed sensitivity for ARMS (49.1%) might appear somewhat lower than in these reports, this could be due to our study design, where blood samples were collected after EGFR-TKI therapy. Indeed, a previous study has reported that the detection rate of *EGFR* mutations in plasma ctDNA from lung cancer patients was lower after chemotherapy or EGFR-TKI therapy [19].

The MassARRAY system is a medium-throughput multiplexed ultrasensitive mutation detection system based on matrix-assisted laser desorption ionization time-of-flight (MALDI-TOF). MassARRAY has been successfully used for the detection of mutations in plasma ctDNA from patients with solid tumors, and the reported limit-of-detection was 0.1% ctDNA [20]. Furthermore, a sensitivity of 61% has been reported using MassARRAY to detect *EGFR* mutations in plasma ctDNA from lung cancer patients [21], while the SABER/MassARRAY method has been successfully used to detect the T790M *EGFR* mutation in EGFR-TKI refractory lung cancer patients [15]. In this study, we further demonstrated that SABER/MassARRAY was a reliable and sensitive method for the detection of exon 19 deletions and L858R *EGFR* mutations in addition to the T790M mutation in plasma ctDNA. Although comparable, the observed sensitivity of SABER/MassARRAY was slightly higher than that of ARMS (56% vs. 49.1%). In our study, SABER/MassARRAY also exhibited higher sensitivity compared to ARMS (50% vs. 33%) for the detection of the T790M mutation associated with acquired EGFR-TKI resistance. In both cases, the trend of higher sensitivity of SABER/MassARRAY, compared to ARMS, could be related to its lower limit-of-detection for mutations in ctDNA (0.1% for SABER/MassARRAY vs. 1% for ARMS) [22]. In our study, false positive results were observed in exon 19 deletions (two patients in ARMS, and one patient in SABER/MassArray). The reasons for positive results may be due to heterogeneity of the tumor, the site of the biopsy, and timing of the biopsy.

In this study, we also found that the presence of *EGFR* mutations in plasma ctDNA detected by ARMS after EGFR-TKI therapy was associated with decreased PFS and OS. Importantly, a previous study also reported a decreased rate of *EGFR* mutations in ctDNA after EGFR-TKI therapy, which was associated with the disease status in lung cancer patients [23]. In our study, the presence of *EGFR* mutations in plasma ctDNA was also associated with a larger tumor size. Therefore, our findings further confirm that the presence of *EGFR* mutations after EGFR-TKI therapy is an adverse prognostic marker in LUAD patients with common *EGFR* activating mutations at diagnosis. In the SABER/MassARRAY group, only trends toward shorter PFS and OS were observed in patients with the presence of EGFR mutations in plasma ctDNA, which may be due to the lower patient number. In addition to ARMS and SABER/MassARRAY, another novel method for the detection of *EGFR* mutations in plasma ctDNA is droplet digital PCR (ddPCR) and next-generation sequencing (NGS) (Table S1). Based on the compartmentalization and amplification of single DNA molecules, ddPCR is generally accepted as the most sensitive method for the detection and quantification of *EGFR* mutations in plasma ctDNA (71%–100% for *EGFR*-activating mutations) [24]. However, in most medical institutions, the use of ddPCR is limited, which is mainly due to the need for specialized equipment and its higher cost in terms of reagents and labor. NGS enables the detection of multiple genetic alterations simultaneously, and has been accepted as a noninvasive tool for the identification and monitoring of cancer mutations. In advanced-stage lung cancer patients, the overall sensitivity of NGS in detecting EGFR mutations from plasma ctDNA has been reported to be between 60.9% and 82.1% [25]. Nevertheless, NGS methods are still relatively expensive and time-consuming [26]. Therefore, our results further demonstrate that, in addition to their use in detecting *EGFR* mutations in tissue samples, both ARMS and MassARRAY currently represent reliable alternatives to ddPCR and NGS for the detection of *EGFR* mutations in plasma ctDNA.

## 5. Conclusions

In conclusion, our study showed that the detection of *EGFR* mutations in plasma ctDNA using either ARMS or SABER/MassARRAY is a promising, minimally invasive, and reliable alternative to tumor biopsy. Moreover, our data indicated that as part of the treatment follow-up, monitoring *EGFR* mutation status in plasma ctDNA could be used as a prognostic marker for LUAD patients carrying common *EGFR*-activating mutations and receiving first-line EGFR-TKI therapy.

## Figures and Tables

**Table 1:** Comparison of amplification refractory mutation system (ARMS) and single allele base extension reaction combined with mass spectroscopy (SABER/MassARRAY) methods in detecting epidermal growth factor receptor (*EGFR*) mutations from circulating tumor DNA (ctDNA).

ARMS vs. Tissue			
ARMS	Tissue		
	Negative	Positive	
Negative	1838.3% RT 90.0% CT 23.4% GT	2961.7% RT 50.9% CT 37.7% GT	
Positive	26.7% RT 10.0% CT 2.6% GT	2893.3% RT 49.1% CT 36.4% GT	
Sensitivity: 49.1%	Specificity: 90%	PPV: 93.3%	NPV: 48.7%
**SABER/MassArray vs. Tissue**			
**SABER/MassArray**	**Tissue**		
	**Negative**	**Positive**	
Negative	1938.3% RT 90.0% CT 23.4% GT	2261.7% RT 50.9% CT 37.7% GT	
Positive	16.7% RT 10.0% CT 2.6% GT	2893.3% RT 49.1% CT 36.4% GT	
Sensitivity: 56%	Specificity: 95%	PPV: 96.6%	NPV: 46.3%

Tissue EGFR was used as the standard reference. PPV: positive predictive value; NPV: negative predictive value.

**Table 2:** Comparison of ARMS and SABER/MassARRAY methods in detecting exon 19 deletions *EGFR* mutations from ctDNA.

Exon 19 deletions ARMS vs. Tissue			
ARMS	Tissue		
	Negative	Positive	
Negative	4338.3% RT 90.0% CT 23.4% GT	1661.7% RT 50.9% CT 37.7% GT	
Positive	26.7% RT 10.0% CT 2.6% GT	1693.3% RT 49.1% CT 36.4% GT	
Sensitivity: 50%	Specificity: 95.6%	PPV: 88.9%	NPV: 72.9%
**SABER/MassARRAY vs. Tissue**			
**SABER/MassARRAY**	**Tissue**		
	**Negative**	**Positive**	
Negative	4338.3% RT 90.0% CT 23.4% GT	1261.7% RT 50.9% CT 37.7% GT	
Positive	16.7% RT 10.0% CT 2.6% GT	1493.3% RT 49.1% CT 36.4% GT	
Sensitivity: 53.8%	Specificity: 97.7%	PPV: 93.3%	NPV: 78.2%

**Table 3:** Comparison of ARMS and SABER/MassARRAY methods in detecting L858R *EGFR* mutations from ctDNA.

L858R ARMS vs. Tissue			
ARMS	Tissue		
	Negative	Positive	
Negative	5738.3% RT 90.0% CT 23.4% GT	1161.7% RT 50.9% CT 37.7% GT	
Positive	06.7% RT 10.0% CT 2.6% GT	993.3% RT 49.1% CT 36.4% GT	
Sensitivity: 45%	Specificity: 100%	PPV: 100%	NPV: 83.8%
**SABER/MassARRAY vs. Tissue**			
**SABER/MassARRAY**	**Tissue**		
	**Negative**	**Positive**	
Negative	5138.3% RT 90.0% CT 23.4% GT	1061.7% RT 50.9% CT 37.7% GT	
Positive	06.7% RT 10.0% CT 2.6% GT	993.3% RT 49.1% CT 36.4% GT	
Sensitivity: 47.4%	Specificity: 100%	PPV: 100%	NPV: 83.6%

Tissue *EGFR* was used as the standard reference. PPV: positive predictive value; NPV: negative predictive value.Tissue *EGFR* was used as the standard reference. PPV: positive predictive value; NPV: negative predictive value.

**Table 4:** Comparison of ARMS and SABER/MassARRAY methods in detecting T790M *EGFR* mutations from ctDNA.

T790M ARMS vs. Tissue			
ARMS	Tissue		
	Negative	Positive	
Negative	7038.3% RT 90.0% CT 23.4% GT	461.7% RT 50.9% CT 37.7% GT	
Positive	06.7% RT 10.0% CT 2.6% GT	293.3% RT 49.1% CT 36.4% GT	
Sensitivity: 33.3%	Specificity: 100%	PPV: 100%	NPV: 94.6%
**SABER/MassARRAY vs. Tissue**			
**SABER/MassARRAY**	**Tissue**		
	**Negative**	**Positive**	
Negative	6438.3% RT 90.0% CT 23.4% GT	361.7% RT 50.9% CT 37.7% GT	
Positive	06.7% RT 10.0% CT 2.6% GT	393.3% RT 49.1% CT 36.4% GT	
Sensitivity: 50%	Specificity: 100%	PPV: 100%	NPV: 95.5%

Tissue *EGFR* was used as the standard reference. PPV: positive predictive value; NPV: negative predictive value.

**Table 5:** Clinical characteristics of first-line EGFR-tyrosine kinase inhibitor (EGFR-TKI) treatment patients.

Characteristics	No (%)
Patient	43 (100)
Sex	
Male	15 (34.9)
Female	28 (65.1)
Smoking	
Yes	4 (9.3)
No	39 (90.7)
Age (year)(median)	71
Pathology	
Adenocarcinoma	43 (100)
EGFR-TKI	
Gefitinib	20 (46.5)
Erlotinib	13 (30.2)
Afatinib	10 (23.3)
Mutations	
Exon 19	27 (62.8)
Exon 21	16 (37.2)
Stage	
IIIB	3 (7)
IV	40 (93)

No: number.

## Supplementary Materials

The following are available online at https://www.mdpi.com/2072-6694/11/6/803/s1. Table S1: Comparison of platforms for detection of *EGFR* mutations from plasma ctDNA; Figure S1: Survival curves showing the progression-free survival (PFS) of lung adenocarcinoma patients with and without detectable epidermal growth factor receptor (*EGFR*) mutations in plasma circulating tumor DNA (ctDNA) by SABER/MassARRAY; Figure S2: Survival curves showing the overall survival (OS) of lung adenocarcinoma patients with and without detectable epidermal growth factor receptor (*EGFR*) mutations in plasma circulating tumor DNA (ctDNA) by SABER/MassARRAY; Figure S3: The average tumor size in patients with detectable plasma *EGFR* mutations compared to those without detectable plasma *EGFR* mutations. A. ARMS, B. SABER/MassARRAY.
