# Supplementary figures and images for: Comparative Analysis of Two Methods for the Detection of EGFR Mutations in Plasma Circulating Tumor DNA from Lung Adenocarcinoma Patients
Source: Cancers (Basel). 2019 Jun 10;11(6):803. doi: 10.3390/cancers11060803 (PMC6627967; doi:10.3390/cancers11060803)

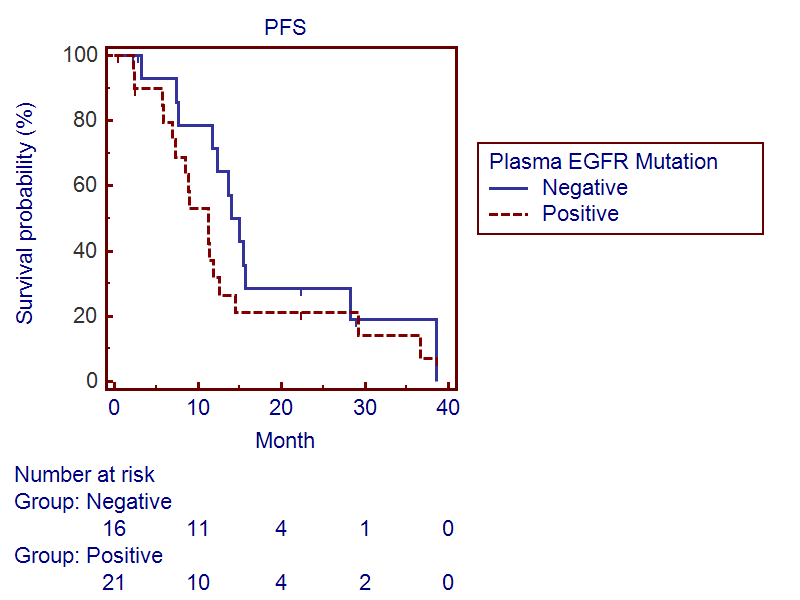

Supplement: Supplementary file 1 [file cancers-11-00803-s001.zip › cancers-508922-supp-online/cancers-508922-supp/Figure S1 PFS.tif]

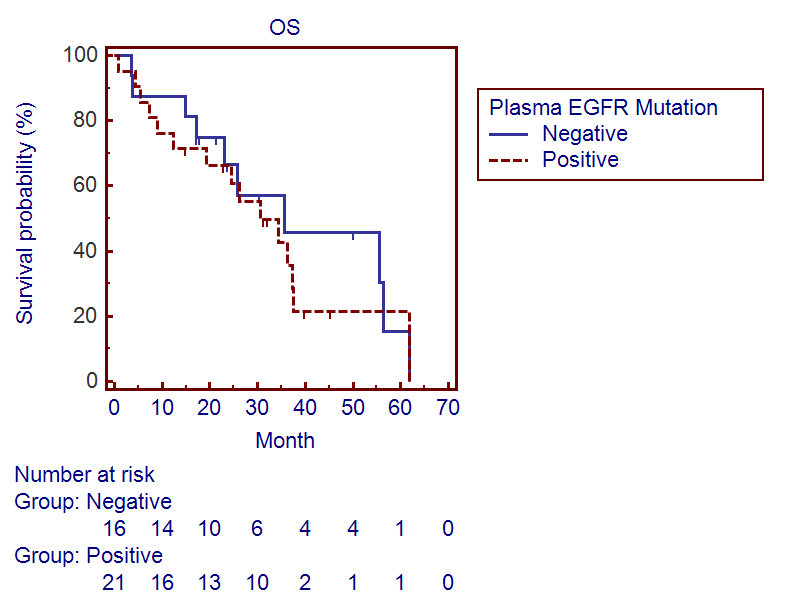

Supplement: Supplementary file 1 [file cancers-11-00803-s001.zip › cancers-508922-supp-online/cancers-508922-supp/Figure S2 OS.tif]

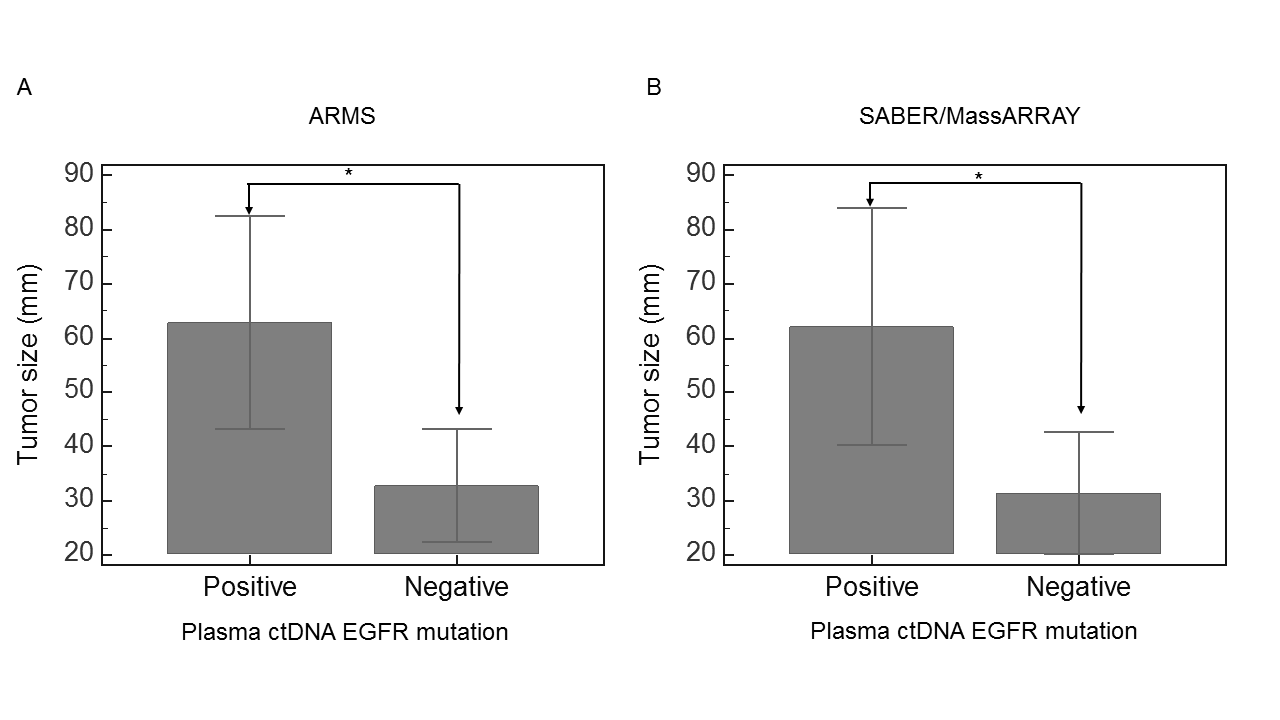

Supplement: Supplementary file 1 [file cancers-11-00803-s001.zip › cancers-508922-supp-online/cancers-508922-supp/Figure S3.tif]
