# Supplementary material for: Comparative Analysis of Two Methods for the Detection of EGFR Mutations in Plasma Circulating Tumor DNA from Lung Adenocarcinoma Patients
Source: Cancers (Basel). 2019 Jun 10;11(6):803. doi: 10.3390/cancers11060803 (PMC6627967; doi:10.3390/cancers11060803)
Supplement: Supplementary file 1 [file cancers-11-00803-s001.zip › cancers-508922-supp-online/cancers-508922-supp/Table S1.doc]

| **Platform** | qPCR | Primer extension  /Mass spectrum | Digital-PCR | NGS |
| --- | --- | --- | --- | --- |
| **Product vendor** | Roche/Qiagen/Amoy | Agena | BioRad/Thermo Fisher/Sysmex | Illumina/Thermo Fisher |
| **Technique** | Real time PCR with fluorescence probe  (Cobas/ARMS) | Single nucleotide extension/Mass Spectrum | Partition of PCR reaction.  Droplet emulsion (Biorad)  Microchip(Thermo Fisher) | Sequencing by synthesis (Illumina)  pH sensor in semiconductor microchip (Thermo Fisher) |
| **Mutation coverage** | Specific known mutations | Specific known mutations | Specific known mutations | Known and unknown mutations |
| **Sensitivity** | 0.5~1% | 1% (0.1% in SABER) | 0.1% (0.01% in Sysmex Beaming) | 0.1% |
| **Turnaround time** | <1 day | 1~2 day | <1 day | 8-10days |
| **Advantage** | Easy to perform  FDA approval  Data comparable between labs | Easy to perform  Mid throughput  Signal could be detected from very short template | Easy to perform  High Dynamic range of detection  Signal could be detected from very short template (Sysmex) | High throughput and high coverage  Identify unknown mutation |
| **Disadvantage** | Detects only known mutations | Detects only known mutations  High instrument cost | Detects only known mutations  High instrument cost (Biorad) | Long turnaround time  Long hand on time |

Table S1. Comparison of platforms for detection of *EGFR* mutations from plasma ctDNA
